# Characterization of a murine xenograft model for contrast agent development in breast lesion malignancy assessment

**DOI:** 10.1186/s12929-016-0261-4

**Published:** 2016-05-17

**Authors:** Tsung-Hsien Yen, Gi-Da Lee, Jyn-Wen Chai, Jiunn-Wang Liao, Jia-Yu Lau, Li-Che Hu, Kuo-Chih Liao

**Affiliations:** Graduate Institute of Biomedical Engineering, National Chung Hsing University, 250 Kuo-Kuang Rd., Taichung City, Taiwan 40227 Republic of China; Department of Radiology, Cheng Ching General Hospital, 118 Sec. 3, Taichung Port Rd., Xitun Dist., Taichung City, Taiwan 40764 Republic of China; Department of Radiology, Taichung Veterans General Hospital, 1650 Sec. 4, Taichung Port Rd., Xitun Dist., Taichung City, Taiwan 40705 Republic of China; College of Medicine, China Medical University, No. 91 Hsueh-Shih Rd., Taichung City, Taiwan 40402 Republic of China; Graduate Institute of Veterinary Pathobiology, National Chung-Hsing University, 250 Kuo-Kuang Rd., Taichung City, Taiwan 40227 Republic of China

**Keywords:** Contrast agent development, Xenograft model, Malignancy screening

## Abstract

**Background:**

The aim of the study was to develop a nude mouse xenograft model implanted with both benign and malignant xenografts as the preliminary candidate screening tool for contrast agent development in lesion malignancy indication.

**Results:**

A malignant xenograft (either MCF-7 cell/matrigel™ or MDA-MB 231 cell/matrigel) and a benign xenograft (culture medium/matrigel) with cleft and slit-like features of intracanaliculer fibroadenoma were implanted subcutaneously into flanks of individual nu/nu nude mouse with >90 % successful inoculation rate. Both malignant and benign xenografts with volume up to 4 cm^3^ and (size up to 2 cm) after 5^th^ week were characterized in vivo by sonogram (exhibiting endogenous morphological contrast features between benign and malignant xenografts), dynamic contrast enhanced multi-detector computed tomography (presenting non-targeting exogenous morphological and dynamic contrast features between benign and malignant xenografts), and then were harvested for histological and immunohistochemistry (revealing example of targeting/molecular contrast features, such as expression of cancer vascular markers of malignant xenografts). Malignant xenografts appeared morphologically taller than wide (axis parallel to skin) with angular/ill-defined margin under sonogram observations, revealed more evident rim enhancement, angular margin and washout pattern in the time-density curve from dynamic contrast enhance multi-detector computed tomography images, and had more visible cancer vascular markers (CD31 and VEGF) expression. With limited number of subjects (5–27 for each group of a specific imaging contrast feature), those imaging contrast features of the xenograft model had larger than 85 % sensitivity, specificity, accuracy, positive and negative prediction values in indicating xenograft malignancy except for results from color Doppler detections.

**Conclusions:**

The murine xenograft model might provide an earlier efficacy evaluation of new contrast agent candidate for lesion malignancy interrogation with qualitative and quantitative indication before a human study to reduce the risk and conserve the resources (time, finance and manpower).

## Background

An important part of breast cancer management is based on the early screening of lesion malignancy and the efficient evaluation of the lesion size/boundary for staging analysis and therapy arrangement [1, 2]. Presently, the invasive needle biopsy procedure is the gold standard in malignancy screening, but it causes discomfort and has the risk of facilitating metastasis due to detached tumor cells caused by the procedure [3–5]. The development of contrast agent enhanced imaging, such as sonogram (US), dynamic contrast enhanced multi-detector computed tomography (DCE-MDCT), magnetic resonance imaging (MRI), and optical imaging etc., which targets the microscopic molecular biomarker discrepancies (such as over expression of epidermal growth factor receptor, EGFR or Erb-1, by cancer cells) [6–9] or reveals the macroscopic anatomical deviations (such as enhanced perfusion from neovascular blood vessel in the cancer tissue) within tumors, benign lesions and normal tissue [10–12], shows promising potentials in both interrogating lesion malignancy and delineating lesion boundaries non-invasively. However, the requirements of adequate target quantities (exceeding minimally topic accumulation concentration of contrast agent and minimally detectable lesion dimension), relatively smaller market value (approximately 1/10 of therapeutic drugs), and equally strict regulations compared to therapeutic agent innovation hamper the development of new imaging agents [13, 14].

The therapeutic value of a leading drug candidate can usually be identified through in vitro studies; however, the feasibility of new contrast agents in specific clinical applications always requires an in vivo model to provide adequate target quantities for verification [13]. To develop an agent for breast lesion malignancy screening, the identification is more challenging; the specificity generally cannot be accessed without human subject studies, because the present animal models do not sufficiently represent key features of clinically benign lesions for screening with contrast agents [15, 16]. The carcinogen 7,12-dimethylbenz[a]anthracene (DMBA) was found to induce both benign and malignant mammary lesions in rats [17]. This DMBA-induced animal model is extensively applied to investigate the mechanism of cancer or the efficiency of preventative agents [18, 19]. However, disadvantages of the model includes modest incidence rate (~60 %), small tumor size (2–55 mm in diameter), and the majority (>90 %) of the DMBA-induced benign masses were glandular neoplasia, which account for less than 10 % of human clinically benign lesions [17]. Fibroadenoma (approximately 45 %) and fibrocystic change (approximately 25 %) are the two major types of clinically benign lesions requiring discrimination from malignant masses [20, 21]. MMTV-c-erbB-2 and MMTV-TGFα transgenic rats can also develop both malignant and benign tumors with over 90 % of those breast abnormalities developing as benign fibroadenomas. The main pitfall of this model is the low transgenic success rate (7–14 %) even with the continued use of the pig follicle stimulating hormone to assist transgene integration into the rat gene sequence [22].

In this study, we are developing a murine xenograft model bearing both malignant and benign xeografts with similar dimension (up to 2 cm) and inoculation position for eliminating the interferences from imaging conditions (discrepancies between subjects due to contrast agent uptake and metabolism differences, or discrepancies between lesions due to relative position difference on subject causing different imaging acquisition conditions) as the preliminary candidate screening tool for contrast agent development in malignancy screening with endogenous morphological, non-targeting exogenous or targeting contrasts. Having both type lesions on the same subject renders possibility to demonstrate the potential of contrast agent candidates in differentiating lesion malignancy between benign and malignant tumors from each imaging result of single subject with both types of lesions, and reduces the required subject number compared to mice with only malignant or benign tumor implant alone in eliminating the interference from discrepancies between subjects for qualitative and quantitative indication of lesion malignancy. Clinically, there were cases with coexistence of both malignant and benign lesion in one subject [23, 24], and there was no evidence suggesting that imaging features (benign or malignant) will be modified by the co-presence of those two types of lesion either in distance or nearby. The xenografts were implanted subcutaneously into the flank region of nude mice (*Foxn1* gene disruption; athymic; nu/nu) for eliminating interferences, such as the accumulation of agents in organs (liver, kidneys, bladder, intestine) responsible for their clearance (stronger contrast enhancement of organs hindering signal from xenograft and resulting in specificity reduction from hindered contrast enhanced indicating malignancy from xenografts’ signal in abdominal area, such as orthotopical breast xenograft), and the unwanted signal interferences from animal hair absorption or scattering [25, 26]. The MDA-MB 231 or MCF-7 cell/matrigel mixture was implanted and allowed to develop to represent the malignant cancer tissue, and the cell culture medium/matrigel mixture was implanted and allowed to stabilize with the surrounding tissue to mimic the solid mass of a benign tumor. The application of matrigel matrix in malignant xenografts has been proven to significantly enhance the grating rate without the requirement of immunosuppressive conditionings (irradiation or medication) before inoculation, while allowing the xenograft to exhibit the histomorphology and molecular markers of cancers [27, 28]. For the benign implant, we created a porous matrigel plug with infiltrated fibrotic cells, instead of mixing benign human breast cell lines (such as MCF-10 or human breast tissue/primary culture) with matrigel matrix, which formed neovasculation (the source of non-specific contrast of conventional contrast enhanced US, DCE-MDCT and MRI that could result in misinterpretation of malignancy of such cell/matrigel benign xenograft) in mice and may evolve into a malignant tumor [27–30]. The two xenografts were then characterized by in vivo imaging inspections (US, CT) to verify the presence of those endogenous morphological and non-targeting exogenous contrasts. Immunohistological analysis of CD31 and VEGF (indications of neovascular development and facilitators for uncontrolled growth, invasion and metastasis of breast cancer [31–33]) in xenograft sections indicated the presence of the endogenous targeting contrasts.

## Methods

### Murine xenograft model for lesion malignancy screening

Nu/nu nude mice (aged 7–9 weeks, 31.3 ± 3.7 g), purchased from BioLASCO Taiwan Co., LTD. (Yilan, Taiwan), were maintained and studied using procedures approved by the Institution Animal Care and Use Committee of National Chung Hsing University (IACUC Approval No. 100–71). Two to three mice were housed to each cage in an individually ventilated, temperature (23 ± 2 °C) and humidity (50–55 %) controlled facilities, on 12 h light, 12 h dark cycle, and had free access to sterilized laboratory chow and water.

The human breast adenocarcinoma cell line, MCF-7 and MDA-MB 231, were obtained from the National Health Research Institute Cell Bank (Hsinchu, Taiwan) and cultured as recommended by the American Type Culture Collection (Manassas, Virginia, USA) with culture reagents obtained from Quantum Biotechnology (distributor of Life Technologies, Inc. and Invitrogen, Taichung, Taiwan) unless otherwise indicated. The cell culture medium was Dulbecco Modified Eagle Medium (DMEM) with 10 % fetal bovine serine (FBS).

Approximately 0.5 ml mixtures (volume ratio = 1:1) of matrigel matrix (Bertec Enterprise Co. Ltd., distributor of BD Bioscience, Taichung, Taiwan) and culture medium (DMEM with 10 % FBS) with or without the suspension of 1 × 10^7^ cancer cells were injected subcutaneously into both dorsal flanks (the same level above the dorsal-ventral adjunction) of the mice to grow xenografts. The mixtures without cells were agitated vigorously to incorporate air bubbles before implantation. The general performance and survival of the mice were monitored twice weekly, and the dimensions of the xenografts (longitudinal length and transverse width) were measured using an electronic digital caliper (Long Jer Precise Industry Co. Ltd., Taichung, Taiwan), and the measurements were applied in calculating the xenograft volume (π/6 × width^2^ × length). The xenografts were allowed to develop for specific durations of time as described below before further characterization with in vivo imaging inspections and histological analysis.

### Sonogram

High resolution sonograms of the xenografts were acquired using a GE Logiq 700 Expert (GE Healthcare, USA) with a 9–12 MHz linear transducer. Before the examination, the mice received an anesthetic agent, Zoletil (zolazepam hypochloride : tiletamine hypochloride = 1 : 1, Virbac Ltd., Milperra, New South Wales, Australia), by intramuscular injection at the dosage of 20–30 mg/kg. The localization and anatomical boundary of the xenografts were investigated first by the gray scale mode detection. The color Doppler measurements were followed to monitor the blood flow in the xenograft.

### DCE-MDCT scan

DCE-MDCT images were acquired using a Philips BR64 (Andover, MA, USA). Before and during the examination periods, anesthesia of the mice was initiated and maintained using the inhalation of isoflurane (Halocarbon, River Edge, NJ, USA) in oxygen (1 % to 2.5 % isoflurane) through a customized mask made specifically for mice. Theclinically approved and well adapted contrast agent, iobitridol (Xenetix, Guerbert, France),was administrated intravenously as a bolus through the tail vein at the clinical dosage of 1 mg/g [34]. Generally, a volume of 100 μl solution at the concentration of 300 mg iodine/ml was injected for a 30 g subject. The images were scanned with the following settings: 0.5 s rotation time, 80 kV, 80 mAs, 0.6 mm beam collimation, and 1 mm slice thickness at different time points after contrast agent administration and were transferred to a PACS workstation (Centricity, GE Medical Systems, Milwaukee, WI, USA) for quantification evaluation. For signal quantification, signal intensity (SI) of xenografts were measured (Hounsfield), and the contrast ratio were calculated as SI_t_/SI_i_, where SI_t_ was the measured intensity at time t and SI_i_ was the measured initial intensity.

### Histology analysis and blood vessel density profile of the xenografts

All of the reagents used in the procedure were purchased from BD Bioscience. The xenografts were harvested after sacrificing the mice, fixed in 10 % buffered formalin, paraffin-embedded, and sectioned. Major organs, such as the liver, lungs, pancreas, kidneys, spleen, heart, intestine and colon were removed and formalin-fixed for further inspection for metastases. The xenograft specimens for histological analysis were processed with conventional hematoxylin and eosin (H&E) staining for the visualization of general tissue morphology and mitotic figures under microscopic inspection. Percentage of neo-vascular blood vessel distributed area in stained xenografts were inspected using a microscope (AF 6000, Leica, Wetzlar, Germany) equipped with a mechanical stage, and linked to a digital color camera (DFC 300, Leica, Wetzlar, Germany) transferring the microscopic images to computer monitor. The presence of neo-vascular blood vessel in xenografts was identified at a magnification of 400×, and the area (same pattern of cell distribution) with neo-vascular blood vessel and boundary of xenografts were marked manually at a magnification of 100× (covering more than 60 % cross-section area of all xenograft images). Percentage of neo-vascular blood vessel distributed area in each xenograft image was calculated by Image J software (1.48v, NIH, USA) with the equation: Area_blood vessel area_ / Area_xenograft_ × 100 %.

### Immunohistochemistry of the xenografts

The immunohistochemistry (IHC) of specimens were stained either with rat-anti-mouse CD31 (1:100 dilution, NCL-CD31-1A10, Novocastra™ Lyophilized Mouse Monoclonal Antibody, Leica Biosystems Newcastle Ltd, UK) or a rabbit polyclonal VEGF antibody (Biorbyt Limited, Cambridge, Cambridgeshire, UK). After deparaffinization and rehydration, formalin-fixed paraffin sections were incubated in 3 % H_2_O_2_ in distilled water for 30 min at room temperature followed by an antigen retrieval step carried out by boiling the slides in 0.01 M of citrate buffer for 20 min. The sections were washed in 50 mM Tris–HCl, 0.05 % Tween, pH 7.6 for 2 min. To block nonspecific binding, all of the sections were treated with 5 % skim milk for 30 min at room temperature. The slides were then incubated with a primary antibody (1:100 dilution for CD31; 1:400 dilution for VEGF) for 2 h at 4 °C. The reaction was stopped by rinsing the section with 0.01 M phosphate-buffered saline (PBS). The slides were then incubated with a biotinylated anti-mouse/rabbit IgG serum (secondary antibody) as a linking reagent, followed by treatment with a peroxidase-labeled streptavidin-biotin complex and diaminobezidine substrate to visualize the positive cells. Finally, the sections were counterstained with hematoxylin prior to being mounted for light microscopy examination.

## Results

**Fig. 1:**
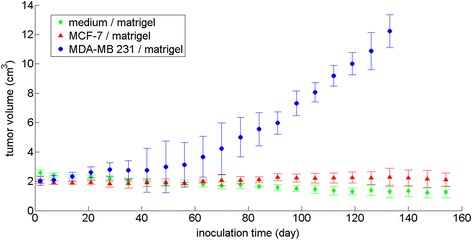
Size of malignant and benign xenografts at time points post implantation. There were 20 MDF-7, 17 MDA-MD 231 and 36 benign xenografts. The xenograft volumes are calculated by dimensions measured with an electronic digital caliper using the following equation: volume = π/6 × width^2^ × length

In the study, 100 % (17/17) of the MDA-MD 231 xenografts, 90.9 % (20/22) of the MCF-7 xenografts and 92.3 % (36/39) of the benign xenografts successfully developed in vivo for image acquisitions and were harvested for histology/IHC analysis. Volume of the benign xenografts reduced gradually (about 2.3 % per week); volume of the MDA-MB 231xenografts had more modest increase (less than 7 % per week) in the initial 6–8 weeks post inoculation and more dramatic growth (about 20 % per week) later; volume of the MCF-7 implants had much mild increase (less than 0.37 %) (Fig. 1) compared to MDA-MB 231 xenografts. No evident metastasis related to the xenograft inoculations was observed with only one subject (with MCF-7 and matrigel xenografts) showing signs of a spleen anomaly at 26 weeks after receiving both benign and malignant implants.

### Histological and immunohistochemical findings of the xenografts

**Fig. 2:**
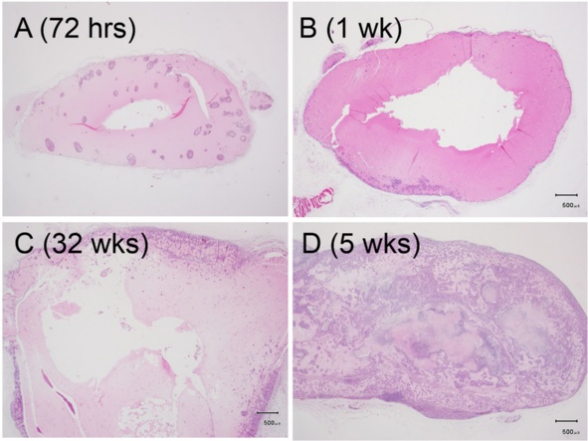
Morphology of MCF-7 xenografts at time points post implantation. The paraffin-embedded, sectioned tissue slices with hematoxylin & eosin staining to visualize morphology were examined by bright field microscopy. **a** Tumor masses were grown as enlarged cysts with numerous adenoid rosette structures with tumor cell groups distributed inside the matrigel wall at 72 h post implantation. **b**, **c** The tumor cells gradually proliferated and aggregated near the exterior edge of the matrigel wall at 1–3 weeks post implantation. **d** The xenografts developed as solid masses with central necrosis after 5 weeks post implantation

**Fig. 3:**
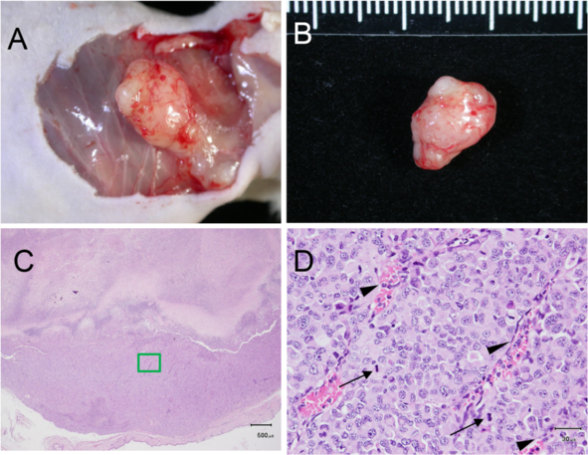
Morphology of fully developed (5-week) MDA-MB 231 xenograft with necrotic core and neovascular blood vessel on the surface or within the mass. There were newly-formed blood vessels on the surface of the developed xenografts identified in situ (**a**) and from the harvested tissue (**b**) and inside the tumor mass with histological H&E staining indicated by black arrow heads (**d**), which was magnified from the green lines marked region in (**c**). The cells with mitotic figures are indicated by black arrows in (**d**)

**Fig. 4:**
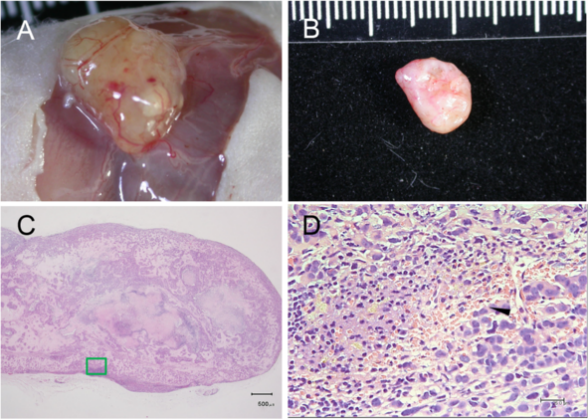
Morphology of fully developed (5-week) MCF-7 xenograft with necrotic core and neovascular blood vessel on the surface or within the mass. There were newly-formed blood vessels on the surface of the developed xenografts identified in situ (**a**) and from the harvested tissue (**b**) and inside the tumor mass with histological H&E staining indicated by arrow heads (**d**), which was magnified from the green lines marked region in (**c**)

**Fig. 5:**
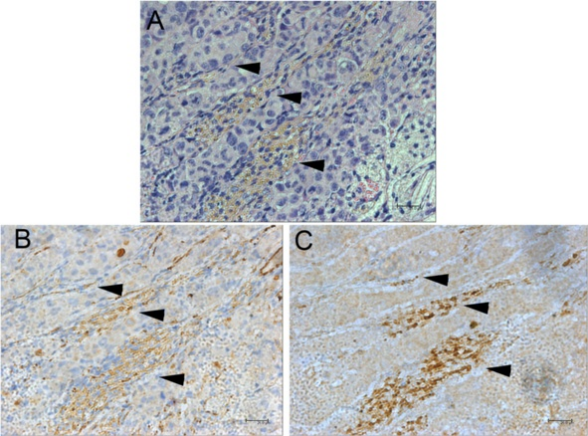
Immunohistochemistry of MDA-MB 231 xenograft. The newly-formed blood vessels appeared inside the tumor mass with histological H&E staining (**a**) and CD31 staining (**b**) indicated by black arrow heads. The VEGF staining exhibited an elevated expression of the cancer pathology-related molecular marker around the region of neovascular vessels indicated by black arrow heads (**c**)

**Fig. 6:**
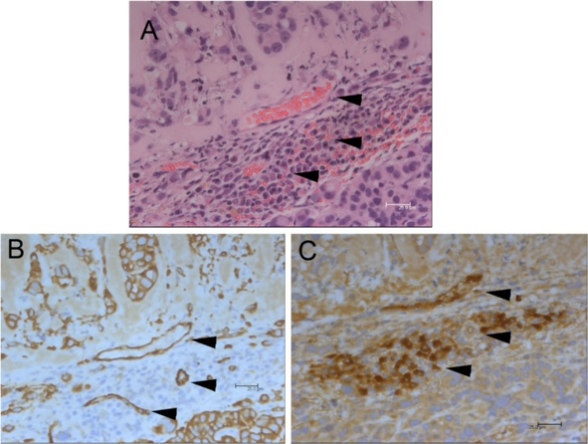
Immunohistochemistry of MCF-7 xenograft. The newly-formed blood vessels appeared inside the tumor mass indicated with H&E staining (**a**) and CD31 staining (**b**) indicated by black arrow heads. The VEGF staining exhibited an elevated expression of the cancer pathology-related molecular marker around the region of neovascular vessels indicated by black arrow heads (**c**)

The bright field microscopic images of the paraffin-embedded, sectioned tissue slices with hematoxylin & eosin staining indicated that MCF-7 tumor masses grew from initial enlarged cysts into solid masses with central necrosis after 5 weeks post implantation (Fig. 2). There were newly-formed blood vessels on the surface of both fully developed malignant xenografts (after 5 weeks post inoculation) identified in situ (Figs. 3a and 4a) and from the harvested tissues (Figs. 3b and 4b), and inside the tumor masses indicated by histological H&E staining (Figs. 5a and 6a) or immunohistochemical CD31 staining (Figs. 5b and 6b). The immunohistochemical VEGF staining showed elevated expression of the cancer pathology-related molecular marker associated with both cell lines [35–38] around region of neovascular vessels (Figs. 5c and 6c).

**Fig. 7:**
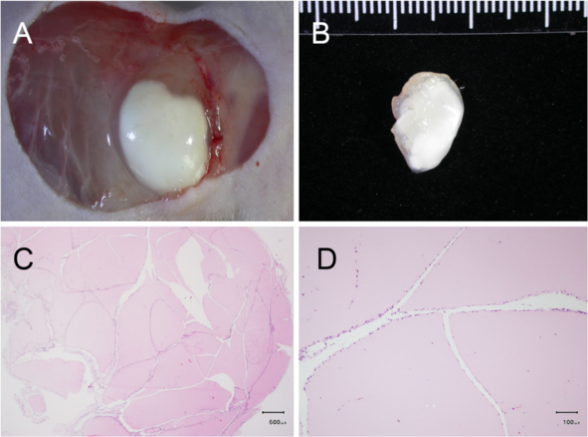
Morphology of the developed (5-week) benign xenograft. There were no noticeable newly-formed blood vessels on the surface of either the xenograft in situ (**a**) or in the harvested tissue (**b**) or inside the developed mass with histological H&E staining (**c**). The benign masses appeared as pink-colored blocks and were infiltrated by fibrotic cells exhibiting cleft- or slit-like openings inside the xenografts (**c**, **d**)

**Fig. 8:**
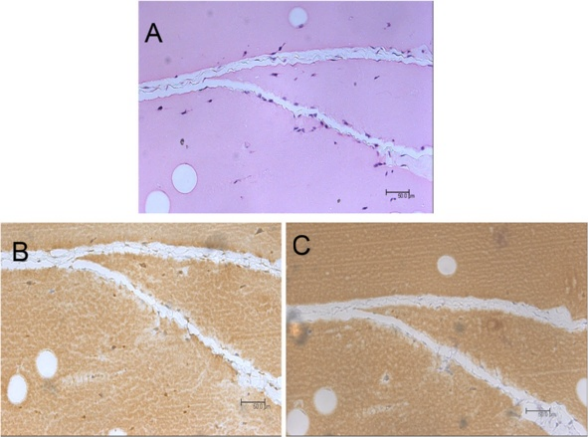
Immunohistochemistry of benign xenograft. There were no noticeable newly-formed blood vessels inside the developed mass with histological H&E staining (**a**) or immunohistochemical CD31 staining (**b**). The immunohistochemical VEGF staining exhibited no evidence of elevated expression inside the xenografts (**c**)

The benign masses appeared as pink-colored blocks under histological H&E staining, and were infiltrated and encapsulated by fibrotic cells exhibiting fibroadenoma-like ductal or slit shaped openings inside the xenografts (Fig. 7c and d) [39, 40]. There were no noticeable newly-formed blood vessels on the surface of either the xenografts in situ (Fig. 7a) or from the harvested tissue (Fig. 7b), or inside the developed mass as indicated by histological H&E staining (Fig. 8a) or immunohistochemical CD31 staining (Fig. 8b). The immunohistochemical VEGF staining showed no evidence of elevated expression inside the xenografts (Fig. 8c).

### Morphology and neovascular blood flow of xenografts under sonogram

**Fig. 9:**
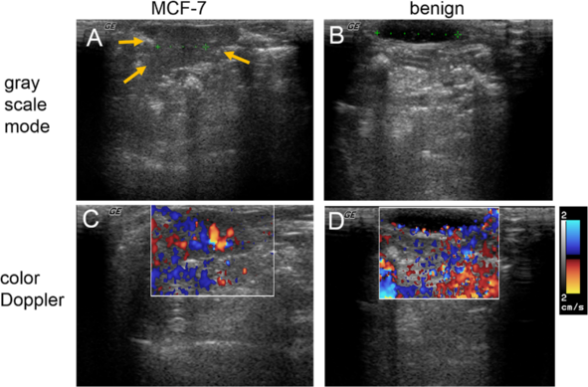
Sonograms and Doppler imaging of developed xenografts in vivo. The longitudinal cross-section images (**a**, **c**) were acquired from malignant xenografts. The transverse cross-section images (**b**, **d**) were acquired from the benign xenografts. The color Doppler imaging (**c**, **d**) quantifies the vascular flow velocity around the xenograft and surrounding region by pseudo colors. The green cursers indicate corresponding xenograft lengths, 0.76 cm in (**a**) and 1.18 cm in (**b**). The morphology of malignant xenograft (MCF-7 xenograft) was demonstrated in (**a**) with angular/ill-defined margin (orange arrows)

Randomly chosen 54 developed xenografts (10 MDA-MB 231, 17 MCF-7 and 27 benign xenogrsfts) were inspected by sonogram. Both malignant xenografts (MDA-MB 231 and MCF-7) grew relatively taller than wide (axis parallel to skin) in orientation with width(axis parallel to skin)/height values from scans with longitudinal orientation of transducer at 1.27 ± 0.25, 1.34 ± 0.31 of MDA-MB 231 and MCF-7 xenografts respectively, compared to 2.94 ± 0.29 of benign xenografts. They also had angular/ill-defined margins (penetrating into adjacent derma layer structures) (Fig. 9a). The detection rate of increased blood flow in the tumor xenografts from color Doppler imaging was 17.6 % (3/17) for MCF-7 xenografts (Fig. 9c) and 10 % (1/10) for MDA-MB 231 xenografts.

The benign masses appeared to have a flat disk-like shape (Fig. 9b). The color Doppler imaging did not show signs of increased blood flow around in any of the benign xenografts (Fig. 9d).

### Morphology and time-density signal profile of xenografts under DCE-MDCT

**Fig. 10:**
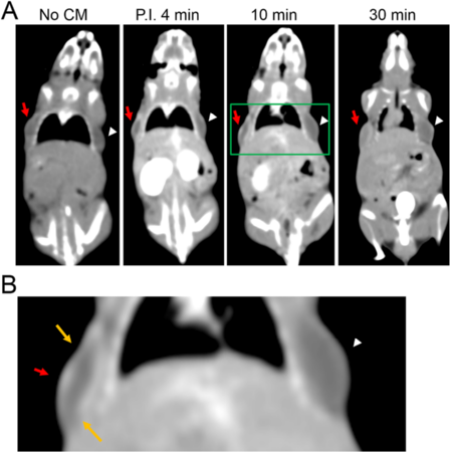
DCE-MDCT of xenografts. The acquisition at time points post iobitridol injection is indicated below each image with the positions of the MDA-MB 231 xenografts indicated by red arrows and the benign xenografts indicated by white arrow heads. **a** The rim enhancement of malignant xenografts (MDA-MB 231) was demonstrated after iobitridol administration. **b** The green lines marked areas in (**a**) are magnified to demonstrate the angular/ill-defined margin (orange arrows) of malignant xenografts (MDA-MB 231)

**Fig. 11:**
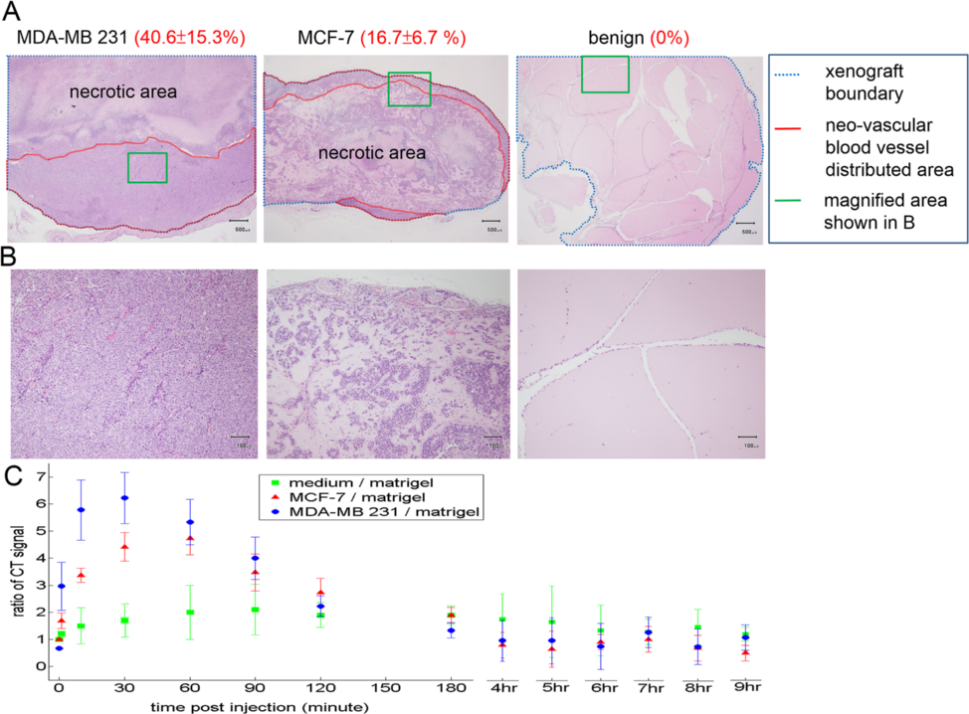
Xenograft type dependent percentage of neo-vascular blood vessel distributed area and time-density profiles under DCE-MDCT. **a** Neo-vascular distributed areas in H&E stained xenografts are marked and quantified at a magnification of 100×. The lesion type dependent percentages of neo-vascular blood vessel distributed area indicated by values in the red parentheses as mean ± standard deviation %. There were 20 MDF-7, 17 MDA-MD 231 and 36 benign xenografts. **b** The green lines marked areas in (**a**) are magnified to demonstrate the xenograft type dependent neo-vascular blood vessel distribution at a magnification of 400×. **c** Time-density profiles of xenografts detected by iobitridol contrasted dynamic CT scan

Randomly chosen 46 developed xenografts (11MDA-MB 231, 12 MCF-7 and 23 benign implants) involved in the DCE-MDCT scan. The average whole body signals (in the unit of Hounsfield) reached peak values in minutes after agent administration and plummeted dramatically, maintaining 50 % of the contrast enhancement for less than 15 min in all of the trials. However, the iobitridol contrasted images indicated that the malignant implants had a more significant contrast enhancement than the benign implants when reaching maximal enhancement (Fig. 10a). MDA-MB 231 xenografts had 620 % maximal contrast enhancement compared to the initial mean value (without contrast medium) approximately 30 min after contrast agent administration and washout-like decline to lose 33.9 % contrast enhancement in the following 60 min with interpolated half-life for 67 min after reaching the peak value (30^th^ minute) or at 97^th^ minute; MCF-7 xenografts had 467 % maximal contrast enhancement approximately 60 min after contrast agent administration and washout-like decline to lose 37 % contrast enhancement in the following 60 min with interpolated half-life for 71 min after reaching the peak value (60^th^ minute) and lasting to 131^st^ minute (Fig. 11c). The benign implants showed a relatively milder maximal contrast enhancement (205 %) in a more extended period (exhibited as a plateau lasting from 60^th^ to 180^th^ minutes). Its interpolated contrast enhancement half-life for 297 min after reaching the peak value (90^th^ minute) and lasting to 387^th^ minute.

The xenograft type dependent features (contrast enhancement maximal values and half-life durations) correlates positively with the xenograft type dependent percentage of neo-vascular blood vessel distributed areas (Fig. 11a and b). MDA-MB 231 xenografts, which had the highest percentage of neo-vascular blood vessel distributed areas at a magnification of 100× (covering more than 60 % cross-section area of all xenograft images), resulted in the most maximal contrast enhancement value and the shortest contrast enhancement half-life duration (Fig. 11c). Benign xenografts, which had no detectable newly-formed blood vessels on the surface of (Fig. 7a and 7b) or within the developed masses (Fig. 11a and b), caused the least maximal contrast enhancement and the longest contrast enhancement half-life duration (Fig. 11c). In examining the morphological features of the xenograft images under DCE-MDCT, the most visible features that could be used to indicate the malignancy of xenografts were the angular/ill-defined margin and rim enhancement. All of the malignant xenografts showed rim enhancement in the initial contrast enhanced period before reaching a peak value with more homogeneous enhancement for the whole xenograft (Fig. 10a). The average enhancement value (Hounsfield) of the rim increased more than 40 % compared to the average value of the central region. Only 8.7 % (2/23) of the benign xenografts showed signs of rim enhancement, while none of them had more than a 25 % increase compared to the average value of the central region. During the same initial contrast enhanced period showing rim enhancement, most of the malignant xenografts (11/11 or 100 % of MDA-MB 231 xenografts; 11/12 or 91.7 % of MCF-7 xenografts) had angular/ill-defined margin, while none of the benign xenografts showed sign of angular/ill-defined margin.

## Discussions

The preliminary results demonstrate that the xenograft model at 5^th^ week (with fully developed anatomical features of lesions and similar sizes between xenografts) exhibits clinical malignancy indicating features of xenografts by endogenous contrasts (taller than wide in orientation, angular/ill-defined margin), non-targeting contrasts (neovascular vessel enhanced perfusion related rim enhancement and time-density patterns), and molecular targeting contrast (cancer marker expression). The xenograft-type occurrence of those contrast features are summarized in Table 1. Corresponding accuracy, sensitivity, specificity, positive predictive value and negative predictive value of those contrast features are shown in Table 2.

**Table 1:** Occurrence of contrast features from the model for malignancy prediction

Contrast features *(Method)*		Xenograft types Occurrence (frequency %)		
		Malignant		Benign
		MDA-MB 231	MCF-7	
Molecular targeting contrast	CD31 *(IHC)*	5/5 (100 %)	6/6 (100 %)	0/11 (0 %)
	VEGF *(IHC)*	5/5 (100 %)	6/6 (100 %)	0/11 (0 %)
Non-targeting contrast	Rim enhancement *(DCE-MDCT)*	11/11 (100 %)	11/12 (100 %)	2/23 (8.7 %)
	Time-density curve			
	Washout T_1/2_ < 100 min *(DCE-MDCT)*	10/11 (90.9 %)	10/12 (83.3 %)	0/23 (0 %)
Endogenous contrast	Angular/ill-defined margin *(grey scale US)*	9/10 (90 %)	14/17 (82.4 %)	0/27 (0 %)
	Angular/ill-defined margin *(DCE-MDCT)*	10/11 (90.9 %)	9/12 (75 %)	1/23 (4.3 %)
	Orientation			
	width/height > 2.2 *(grey scale US)*	10/10 (100 %)	17/17 (100 %)	0/27 (0 %)
	Neovascular flow *(color Doppler US)*	1/10 (10 %)	3/17 (17.6 %)	0/27 (0 %)

Occurrence = occurred number of specific type xenograft/total number of specific type xenograft; frequency % = occurred number of specific type xenograft/total number of specific type xenograft × 100 %

**Table 2:** Reliability of contrast features from the model for malignancy prediction

Contrast features *(Method)*		Accuracy	Sensitivity	Specificity	PPV	NPV
Molecular targeting contrast	CD31 *(IHC)*	100 %	100 %	100 %	100 %	100 %
	VEGF *(IHC)*	100 %	100 %	100 %	100 %	100 %
Non-targeting contrast	Rim enhancement *(DCE-MDCT)*	93.5 %	95.7 %	91.3 %	91.7 %	95.5 %
	Time-density curve	91.3 %	86.9 %	100 %	100 %	88.5 %
	Washout T_1/2_ < 100 min *(DCE-MDCT)*					
Endogenous contrast	Angular/ill-defined margin *(grey scale US)*	92.6 %	85.2 %	100 %	100 %	100 %
	Angular/ill-defined margin *(DCE-MDCT)*	93.5 %	87 %	95.7 %	95.2 %	87 %
	Orientation width/height > 2.2 *(grey scale US)*	100 %	100 %	100 %	100 %	100 %
	Neovascular flow *(color Doppler US)*	57.4 %	14.8 %	100 %	100 %	54 %

TP_MDA_ = number of true positive (indicating malignancy) of MDA-MB 231 xenografts; TP_MCF_ = number of true positive (indicating malignancy) of MCF-7 xenografts; TN = number of true negative (not indicating malignancy) of benign xenografts; FP = number of false positive (indicating malignancy) of benign xenografts; FN_MDA_ = number of false negative (not indicating malignancy) of MDA-MB 231 xenografts; FN_MCF_ = number of false negative (not indicating malignancy) of MCF-7 xenografts; accuracy = (TP_MDA_ + TP_MCF_ + TN)/(TP_MDA_ + TP_MCF_ + FN_MDA_ + FN_MCF_ + TN + FP) × 100 %; sensitivity = (TP_MDA_ + TP_MCF_)/(TP_MDA_ + TP_MCF_ + FN_MDA_ + FN_MCF_); specificity = TN/(TN + FP); PPV (positive predictive value) = (TP_MDA_ + TP_MCF_)/(TP_MDA_ + TP_MCF_ + FP); NPV (negative predictive value) = (TN)/(FN_MDA_ + FN_MCF_ + TN); washout T_1/2_ = half-life of washout-like contrast pattern

With limited number of subjects (5–27 for each contrast feature analysis compared to clinical studies with much larger than 100 [15, 16]), each occurrence of false positive (indicating malignancy for benign xenograft) or false negative (not indicating malignancy for malignant xenograft) greatly reduce accuracy, sensitivity, specificity, positive predictive value and negative predictive value by 1.8–5 %. However, most of the contrast features achieved exceeding 85 % accuracy, sensitivity, specificity, positive predictive value and negative predictive value except for the contrast from neovascular blood flow detected by color Doppler US. Those contrast features of the xenograft model with high (exceeding 85 %) accuracy, sensitivity, specificity, positive predictive value and negative predictive value, surpassing the evaluation efficiency from 3 conventional imaging modalities (mammogram had 70.2 % accuracy, 67.8 % sensitivity, 75 % specificity and 85.7 % positive predictive value; US had 67.8 % accuracy, 83 % sensitivity, 34 % specificity and 73.5 % positive predictive value; MRI had 72.9 % accuracy, 94.4 % sensitivity, 26 % specificity and 73.6 % positive predictive value) for breast lesion screening [15]. It verifies that the xenograft model can be applied as the preliminary candidate screening tool for contrast agent development in lesion malignancy indication with reduced number of subjects, because bearing both malignant and benign xenografts on the same subject with similar size and inoculation position can eliminate the interferences from imaging conditions (discrepancies between subjects due to contrast agent uptake and metabolism differences, or discrepancies between xenografts due to relative position difference on subject causing different imaging acquisition conditions).

The neovascularity related blood flow detected by color Doppler US was the only inconclusive contrast feature of the xenograft model in indicating the malignancy of malignant xenografts with low occurrence frequency of true positive events (17.6 %, 3/17, for MCF-7 xenografts and 10 %, 1/10, for MDA-MB 231 xenografts) and correspondingly poor accuracy (57.4 %), sensitivity (14.8 %) and negative predictive value (54 %). One of the potential reason is that the application of probe pressure caused deformation of blood vessel, obstruction of the vascular blood flow and therefore the malignant xenografts appeared to resemble an avascular xenograft/lesion [41–43]. Another explanation could be the limited capability of Doppler-based imaging to detect low velocity (<1 cm/s) blood flow from small blood vessels of xenograft neovascularity [44].

For conventional DCE-MDCT for lesion malignancy screening, angular/ill-defined margin (99–100 %) and rim enhancement (100 %) are the most predictive CT clinical features of breast lesion malignancy with high accuracy, while a washout pattern on postcontrast images has high sensitivity (91 %) on positive malignant lesions but low specificity (48 %) with high false positive occurrence rate (42 %) [45, 46]. Clinically, washout pattern in conventional DCE-MDCT have abruptly decline happened in 10 min after contrast medium injection [47], however the half-life of washout-like pattern from malignant xenografts of the xenograft model detected by DCE-MDCT approached 70 min. The potential reason for the prolonged washout-like pattern is that the subcutaneous xenografts were inoculated in the artificial flank area for the advantage of reducing interference from major organs (which are responsible for contrast agent excretion and accumulate with them, or have higher blood vessel densities to cause higher contrast enhancement and hinder the signal from xenografts) and the corresponding disadvantage with less interaction with central circulation (less purfusion) as the actual malignant lesion [48].

From the preliminary detection results of the two available clinical medical imaging modalities, US appropriately indicated endogenous morphological contrast features between benign and malignant xenografts of the animal model associated with density/echogenicity deviation of lesion tissue, while DCE-MDCT adequately presented non-targeting exogenous morphological and dynamic contrast features between benign and malignant xenografts related to regionally time-dependent or neovascular density dependent contrast agent distribution.

The developing xenograft model for contrast agent screening had limitations. Both types of xenografts were not orthotopic lesions, which might not fully recapitulate the characteristics of actual lesions in human breast due to lack of micro-cellular environment mimicking human breast gland with stromal component of tumor origin, lower level exposure of systematic hormones and growth factors from mice than human [49, 50]. Results obtained from the models cannot precisely predict the clinical sensitivity, specificity, accuracy, positive and negative prediction values of the screened contrast agent candidate in actual medical use. However, those xenografts had mimicking 3 days structure of benign and malignant lesions, endogenous targeting features or exogenous non-targeting features for potential contrast agents, and allowed quick indication of contrast agent potential in malignancy assessment by eliminating discrepancies between subjects and revealing contrast agent potential qualitatively and quantitatively in each imaging acquisition.

## Conclusions

In conclusion, the xenograft model exhibiting clinical endogenous, non-targeting exogenous, and molecular targeting contrasts, has the potential to be applied as the low cost preliminary screening tool for testing contrast agents candidates of conventional imaging or molecular imaging (except for color Doppler contrast agents) with the advantage to inoculate both benign and malignant xenografts on the same subject and eliminate the interference from difference between individual subjects, and to reduce the required subject numbers. This model might provide an earlier efficacy evaluation of new contrast agent candidate for lesion malignancy interrogation before a human study to reduce the risk and conserve the resources (time, finance and manpower).

## Abbreviations

DCE-MDCT: Dynamic contrast enhanced multi-detector computed tomography
DMBA: 7,12-dimethylbenz[a]anthracene
DMEM: Dulbecco’s Modified Eagle Medium
FBS: Fetal bovine serine
H&E: Hematoxylin and eosin
IHC: Immunohistochemistry
MMTV: Mouse mammary tumor virus
MRI: Magnetic resonance imaging
TGFα: Transforming growth factor alpha
US: Sonogram
VEGF: Vacular endothelial growth factor
